# Macrophage nuclear receptors: Emerging key players in infectious diseases

**DOI:** 10.1371/journal.ppat.1007585

**Published:** 2019-03-21

**Authors:** Chrissy M. Leopold Wager, Eusondia Arnett, Larry S. Schlesinger

**Affiliations:** Texas Biomedical Research Institute, San Antonio, Texas, United States of America; Geisel School of Medicine at Dartmouth, UNITED STATES

## Abstract

Nuclear receptors (NRs) are ligand-activated transcription factors that are expressed in a variety of cells, including macrophages. For decades, NRs have been therapeutic targets because their activity can be pharmacologically modulated by specific ligands and small molecule inhibitors. NRs regulate a variety of processes, including those intersecting metabolic and immune functions, and have been studied in regard to various autoimmune diseases. However, the complex roles of NRs in host response to infection are only recently being investigated. The NRs peroxisome proliferator-activated receptor γ (PPARγ) and liver X receptors (LXRs) have been most studied in the context of infectious diseases; however, recent work has also linked xenobiotic pregnane X receptors (PXRs), vitamin D receptor (VDR), REV-ERBα, the nuclear receptor 4A (NR4A) family, farnesoid X receptors (FXRs), and estrogen-related receptors (ERRs) to macrophage responses to pathogens. Pharmacological inhibition or antagonism of certain NRs can greatly influence overall disease outcome, and NRs that are protective against some diseases can lead to susceptibility to others. Targeting NRs as a novel host-directed treatment approach to infectious diseases appears to be a viable option, considering that these transcription factors play a pivotal role in macrophage lipid metabolism, cholesterol efflux, inflammatory responses, apoptosis, and production of antimicrobial byproducts. In the current review, we discuss recent findings concerning the role of NRs in infectious diseases with an emphasis on PPARγ and LXR, the two most studied. We also highlight newer work on the activity of emerging NRs during infection.

## Introduction

Nuclear receptors (NRs) comprise a superfamily of intracellular transcription factors that are key players in macrophage homeostasis, metabolism, and transcriptional regulation [1–5]. NRs are activated upon interaction with specific ligands, which are typically lipid-soluble and membrane-permeable. Macrophages use NRs to sense their local environment, shaping their immune response. There are 48 NRs in the human genome [6] and 49 in the rodent genome, of which 28 are associated with macrophages [7] (see Table 1 for selected members). Members of the NR superfamily have historically been categorized into three classes: conventional steroid/thyroid hormone receptors (e.g., estrogen receptor, progesterone receptor), orphan receptors for which the ligand has either not been identified or that appear to function without a ligand, and adopted orphan receptors for which a ligand has been discovered (e.g., liver X receptors [LXRs], peroxisome proliferator-activated receptors [PPARs], and retinoid X receptors [RXRs]) [8].

**Table 1:** Select NR family members. List of some commonly studied NRs.

Classification	Group	Abbreviation	NRNC symbol	Gene	Endogenous ligands
**Steroid hormone receptors**	Thyroid hormone receptor	THRα	NR1A1	*THRA*	Thyroid hormones
		THRβ	NR1A2	*THRB*	
	Retinoic acid receptor	RARα	NR1B1	*RARA*	Retinoid acids
		RARβ	NR1B2	*RARB*	
		RARγ	NR1B3	*RARG*	
	Glucocorticoid receptor	GR or GCR	NR3C1	*NR3C1*	Glucocorticoids
	Vitamin D receptor	VDR*	NR1I1	*VDR*	Vitamin D
**Adopted orphan receptors**	Peroxisome proliferator-activated receptor	PPARα*	NR1C1	*PPARA*	Fatty acids
		PPAR β/δ*	NR1C2	*PPARD*	
		PPARγ*	NR1C3	*PPARG*	
	REV-ERB	REV-ERBα*	NR1D1	*NR1D1*	Heme
		REV-ERBβ	NR1D2	*NR1D2*	
	Liver X receptor	LXRα*	NR1H3	*NR1H3*	Oxysterols
		LXRβ*	NR1H2	*NR1H2*	
	Farnesoid X receptor	FXR*	NR1H4	*NR1H4*	Bile acids
	Pregnane X receptor	PXR*	NR1I2	*NR1I2*	Xenobiotics
	Retinoid X receptor	RXRα	NR2B1	*RXRA*	9-*cis* retinoic acid
		RXRβ	NR2B2	*RXRB*	
		RXRγ	NR2B3	*RXRG*	
**Orphan receptors**	RAR-related orphan receptor	RORα	NR1F1	*RORA*	—
		RORβ	NR1F2	*RORB*	
		RORγ	NR1F3	*RORC*	
	Testicular receptor	TR2	NR2C1	*NR2C1*	
		TR4	NR2C2	*NR2C2*	
	Estrogen-related receptor	ERRα*	NR3B1	*ESRRA*	
		ERRβ	NR3B2	*ESRRB*	
		ERRγ*	NR3B3	*ESRRG*	
	NR4A family	Nur77*	NR4A1	*NR4A1*	
		Nurr1*	NR4A2	*NR4A2*	
		Nor1*	NR4A3	*NR4A3*	
	Liver receptor homolog-1	LRH-1*	NR5A2	*NR5A2*	

*NRs recently shown to influence macrophage responses to pathogenic microbes that are discussed in this review. Some synthetic and/or endogenous ligands have been suggested for certain orphan receptors, however they have not yet been confirmed.

**Abbreviations:** NR, nuclear receptor; NRNC, nuclear receptor nomenclature committee; NR4A, nuclear receptor 4A; RAR, retinoic acid receptor.

In the United States, approximately $70 billion was spent to treat infectious diseases in 2013, and the cost per case for treatment grew the fastest compared to other diseases between 2000 and 2013 [9]. With the dramatic increase in the prevalence of pathogens becoming resistant to existing therapeutics that target the microorganism (antibiotics, antivirals, antiparasitics, etc.), it is critical to consider novel host-directed therapeutics (HDTs) to assist in combating infectious diseases. Approximately 13% of drugs approved for sale in the US target NRs and represented $27.5 billion in sales revenue in 2009 [10]. Drugs targeting NRs are typically used to treat diabetes, atherosclerosis, and autoimmune diseases. However, NRs are becoming increasingly appreciated in the context of infections; therefore, targeting NRs may provide a new, largely unexplored area for infectious disease treatment.

## NRs: Structure and function

**Fig 1:**
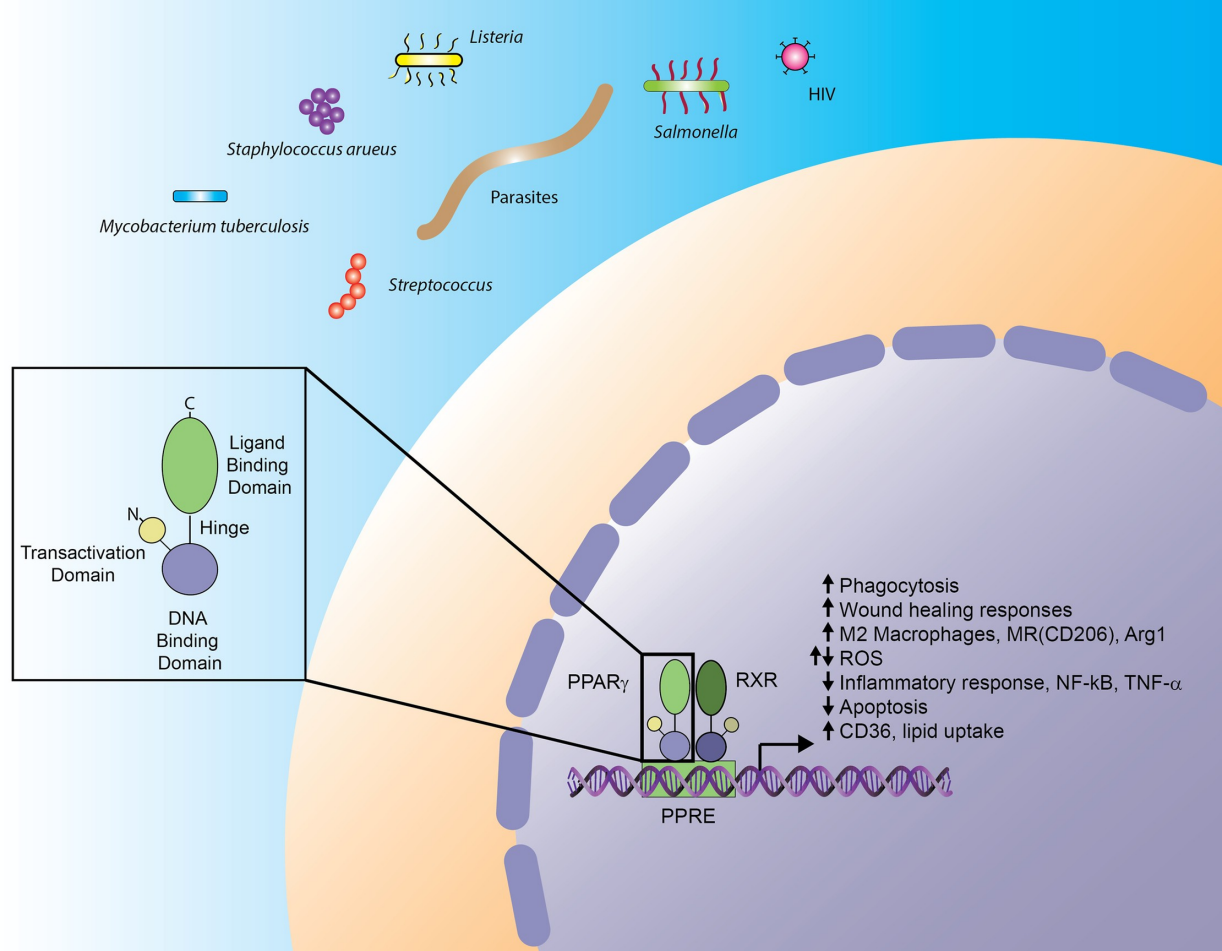
PPARγ structure and signaling in macrophages. NRs have a highly conserved structure with an N-terminal transactivation domain and a DNA-binding domain and C-terminal ligand-binding domain that are connected by a hinge region. Upon activation, the NR PPARγ heterodimerizes with RXR, binds to PPREs, and initiates transcription of target genes initiating various signaling pathways in macrophages. PPARγ signaling is induced in macrophages following infection with several pathogens, including bacteria, parasites, and viruses. During infection, PPARγ signaling has been linked to increased wound-healing responses, M2 macrophages, phagocytosis, and decreased inflammation. PPARγ has also been linked to decreased apoptosis and increased lipid uptake in macrophages. Conversely, PPARγ has been linked to both reduced and increased ROS production, including increased mitochondrial ROS production in the absence of NADPH oxidase. Arg1, arginase 1; CD36, cluster of differentiaion 36; MR, mannose receptor; NADPH, nicotinamide adenine dinucleotide phosphate; NF-κb, nuclear factor kappa B; NR, nuclear receptor; PPARγ, peroxisome proliferator-activated receptor gamma; PPRE, PPAR response element; ROS, reactive oxygen species; RXR, retinoid X receptor; TNF-α, tumor necrosis factor α.

NRs have a common architecture, containing an N-terminal transactivation domain, a highly conserved DNA-binding domain and a carboxy-terminal ligand-binding domain (reviewed in [11], Fig 1). NRs are activated following binding to the receptor’s specific ligand. The NR then undergoes a conformational change which can lead to recruitment of coactivators and/or corepressors [12]. NRs can exist as monomers, homodimers, or heterodimers (often with RXR) and can positively regulate transcription by binding to specific response elements of their target genes. NRs can also regulate transcription by repression or *trans*-repression [3–5] and inhibition of other transcription factors like nuclear factor kappa B (NF-kB) (reviewed in [8]). NR activity and stability can be further regulated by post-translational modifications (i.e., phosphorylation, acetylation, sumoylation, and ubiquitination) [13].

## NRs and macrophage inflammatory responses

Macrophages have numerous, diverse roles in the host and are typically regarded as capable of activating to a pro-inflammatory, antimicrobial phenotype or an anti-inflammatory, wound-healing phenotype that does not efficiently kill microbes [14–17]. However, macrophage activation is now viewed as a spectrum rather than two distinct polarization states (e.g., M1 or M2), and many macrophages exhibit a mixed phenotype dependent on numerous factors in the local tissue environment [14, 18–24].

NRs are intimately involved in macrophage development as well as in macrophage inflammatory response and host defense pathways. PPARγ repeatedly appears as a regulator of alveolar macrophage (AM) development and polarization [25–28], and LXRα is required for maturation of Kupffer cells (liver-resident macrophages) and differentiation of macrophages in the marginal zone of the spleen [29, 30]. In AMs, PPARγ is constitutively expressed at high levels, contrasting with its expression in blood and other tissue macrophages [28, 31, 32]. PPARγ expression in AMs is important for inhibiting Th1-type pro-inflammatory responses, resolution of inflammation, and for maintaining homeostasis in murine lungs [28, 33]. PPARγ is also important for expansion of hematopoietic stem cells from cord blood in humans but not in mice [34]. The Th2-associated cytokine interleukin-4 (IL-4) augments PPARγ expression, induces the generation of PPARγ ligands, and contributes to the maturation of M2 macrophages [35, 36]. Recent studies have identified protein arginine methyltransferase 1 (PRMT1) as a critical enzyme for PPARγ’s responsiveness to IL-4 due to the asymmetric demethylation of histone H4 arginine 3 at the PPARγ promoter [37]. This study also revealed PRMT1’s importance for the transition from monocyte to macrophage. A recent study showed that PPARγ is recruited to the genome in a ligand-independent manner during polarization with IL-4 in murine macrophages [38]. Upon repeated IL-4 exposure, ligand-insensitive PPARγ established a permissive chromatin environment that conferred transcriptional memory preferential to the M2 polarization state and resistance to IFN-γ stimulation [38]. This study revealed a PPARγ-mediated mechanism for memory-like responses in macrophages independent of ligand binding.

Several other NRs play significant roles in macrophage inflammatory responses. For example, many genes inhibited by LXR agonists are targets of NF-kB [39], indicating an inhibitory effect of LXR on pro-inflammatory responses. REV-ERBα is expressed in primary human macrophages and is induced by LXR [40]. REV-ERBα is a constitutive repressor that is more highly expressed in M1- compared to M2-activated human macrophages, repressing IL-10 production [41]. In murine macrophages, a REV-ERBα agonist suppressed transcription of IL-6 and C-C motif chemokine ligand 2 (CCL-2), as well as the CCL-2 activated signals extracellular signal-regulated kinase (ERK) and p38 [42, 43], indicating that this NR’s anti-inflammatory actions are conserved between species. Another family of NRs, the NR4A family, is also expressed in several macrophage models, and family members are up-regulated in response to inflammatory stimuli such as lipopolysaccharide (LPS) or tumor necrosis factor α (TNF-α), promoting anti-inflammatory, M2-like macrophage responses [44–49]. NR regulation of macrophage inflammatory responses are reviewed in more detail elsewhere [1, 4, 5, 7, 8, 50]. In this review, we discuss recent literature regarding NRs shown to influence macrophage responses in infectious diseases.

## PPARs

**Fig 2:**
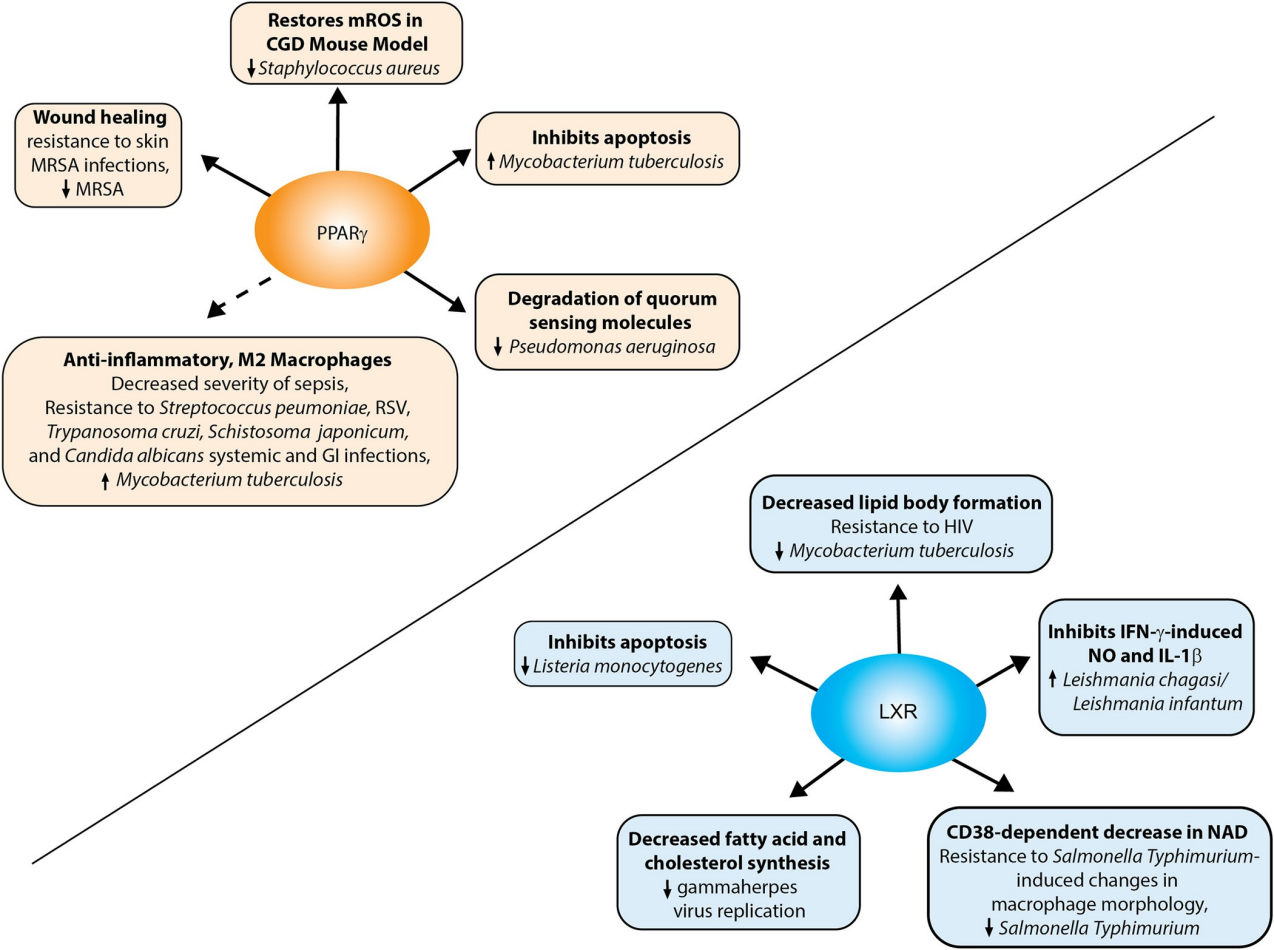
PPARγ and LXR signaling in macrophages results in resistance or susceptibility to pathogens. Infection with different pathogens can result in activation of PPARγ and LXR. Pathways regulated by these NRs and their impact on the host (mediating a resistant or susceptible phenotype) and pathogen (altered growth specified by ↑ or ↓) are indicated. Solid arrows indicate NR-mediated mechanisms between macrophage responses and effect on pathogen and/or disease. The dashed arrow indicates correlative links between NR-mediated macrophage responses and disease. CGD, chronic granulomatous disease; GI, gastrointestinal; LXR, liver X receptor; MRSA, methicillin-resistant *Staphylococcus aureus*; NAD, nicotinamide adenine dinucleotide; NO, nitric oxide; PPARγ, peroxisome proliferator-activated receptor gamma; RSV, respiratory syncytial virus.

PPAR activities in macrophages are important for the control of transport, synthesis, activation, and oxidation of fatty acids [2]. Mammals have three PPAR subtypes: PPARα, PPARγ, and PPARβ/δ (also referred to as NR1C1, NR1C3, and NR1C2, respectively; Table 1). The subtypes exhibit diverse expression patterns and functions. PPARα and PPARγ are expressed in macrophages and are important players in macrophage inflammatory responses [36, 51, 52]. Earlier studies examining the complex relationship of PPARs and infectious diseases are reviewed elsewhere [1, 53, 54]. Herein, we focus on the more recently elucidated roles of PPARs in macrophage responses (Figs 1 and 2).

### Bacteria

As discussed above, PPARγ signaling is important for the development of AMs in the lung as well as promotion of anti-inflammatory macrophages and mitigation of inflammation-induced host damage [25, 26, 28, 33]. In the case of *Streptococcus pneumoniae* infection, PPARγ was critical for dampening the inflammatory response in murine lungs, demonstrating its importance for maintaining lung function and the local lung macrophage population and reducing host mortality [28]. PPARγ is also critical for clearance of methicillin resistant *Staphylococcus aureus* (MRSA) in skin infections. MRSA was able to resist the inflammatory response but succumbs to the resolving abscess microenvironment, which is replete with antimicrobial peptides [55]. Myeloid cell PPARγ signaling was essential for maintaining the resolution phase of MRSA skin infections in mice, and activation of PPARγ hastened the onset of wound resolution [55]. Treatment with the PPARγ agonist pioglitazone in a mouse model of chronic granulomatous disease (CGD; NADPH deficiency) with *S*. *aureus* infection restored reactive oxygen species (ROS) production by mitochondria in blood monocytes and tissue macrophages [56]. These results were replicated in monocytes from human CGD patients, demonstrating that PPARγ activation appears to bypass the need for NADPH oxidase activation, partially restoring host defense against *S*. *aureus* in CGD [56]. Activation of PPARγ was also conducive to macrophage killing of *Pseudomonas aeruginosa*, likely through degradation of the bacterium quorum–sensing molecules, which are able to decrease PPARγ expression [57].

In contrast to the activities described above, PPARγ is important for *Mycobacterium tuberculosis* replication [58–60]. Recent studies revealed that macrophages in the lungs of PPARγ knock-out mice, but not peritoneal macrophages or bone marrow derived macrophages (BMDMs), are more resistant to *M*. *tuberculosis* growth ex vivo [61]. PPARγ deficiency also resulted in increased pro-inflammatory cytokine production in the lungs of *M*. *tuberculosis-*infected mice, correlating with decreased bacterial burden [61]. Another recent study demonstrated that vitamin B1 can inhibit PPARγ expression, promoting NF-kB signaling and resulting in macrophage control of *M*. *tuberculosis* [62]. Conversely, PPARα is reported to be essential for antimycobacterial responses by activating autophagy, lipid catabolism, and fatty acid oxidation [52]. Agonists of PPARα promoted autophagy, lysosomal biogenesis, phagosome maturation, and defended against *M*. *tuberculosis* and *Mycobacterium bovis* bacillus Calmette-Guerin (BCG) in BMDMs [52], demonstrating an important role for PPARα in host defense against mycobacteria.

Less virulent mycobacteria either do not elicit PPARγ expression or induce expression to a lesser extent than virulent strains [58, 63, 64], suggesting that virulent *M*. *tuberculosis* has evolved to select for induction of PPARγ-mediated pathways in order to alter the environment and enhance its growth. Furthermore, *M*. *tuberculosis* and *M*. *bovis* BCG induction of PPARγ signaling is linked to ligation of the pattern recognition receptors (PRRs) mannose receptor (MR; CD206) and toll-like receptor 2 (TLR2), respectively [58, 63, 65]. PPARγ activity in macrophages requires the enzymes cytosolic phospholipase A_2_ (cPLA_2_) and 15-lipoxygenase (15-LOX), which are important for the production of inflammatory lipid-signaling molecules, termed eicosanoids, that are linked to both *M*. *tuberculosis* resistance and susceptibility [58, 66, 67]. Recently, our laboratory has identified several genes regulated by PPARγ during *M*. *tuberculosis* infection of human macrophages, including cyclooxygenase-2 (COX-2), S100A8, and CMKLR1, which are all linked to eicosanoid production [66]. We also demonstrate that PPARγ induction of the antiapoptotic protein Mcl-1 during *M*. *tuberculosis* infection inhibits macrophage apoptosis [66]. Inhibition of Mcl-1 as well as 15-LOX significantly reduced *M*. *tuberculosis* growth. Altogether, these data indicate that PPARγ is involved in regulating pathways critical for *M*. *tuberculosis* survival, including apoptosis and the production of lipid mediators. These findings support the idea that *M*. *tuberculosis* modifies macrophage signaling processes to promote its own survival, which is particularly relevant in AMs.

### Viruses

M2 macrophages are important for negating damaging inflammation. In a respiratory syncytial virus (RSV) model, PPARγ induced M2 development, decreasing lung pathology [68]. Treatment with the PPARγ agonist rosiglitazone also aided in skewing the macrophages to an M2 phenotype. Patients with human immunodeficiency virus (HIV) infection had decreased PPARγ gene and protein expression in AMs, correlating with increased oxidative stress [69]. Ex vivo treatment with a PPARγ agonist increased PPARγ levels, improved the phagocytic index of the AMs, and decreased oxidative stress [69], thus supporting the potential role of PPARγ agonists for treatment. Monocytes from HIV patients showed reduced expression of not just PPARγ but also PPARα, RXR, PXR, and FXR, which was not restored by highly active antiretroviral therapy (HAART) [70]. Conversely, LXR expression was up-regulated in these patients. This study revealed a correlation between HIV patients’ lipid and cholesterol abnormalities and decreased expression of PPARγ and the cell surface receptor CD36 [70]; the latter aids in uptake of oxidized low-density lipoproteins by monocytes as well as the major surfactant lipid dipalmitoyl phosphatidylcholine [71].

### Sepsis

Sepsis models have revealed a critical role for PPARγ in controlling inflammation and improving the overall disease outcome. PPARγ gene expression was down-regulated in freshly isolated peripheral blood mononuclear cells (PBMCs) from human sepsis patients [72] as well as in the lungs in a murine sepsis model [73]. In mice, this down-regulation correlated with increased plasma levels of TNF-α, IL-6, and IL-10. Contrary to expectations, peritoneal macrophages from PPARγ knock-out mice released less IL-1α and IL-1β as a result of increased IFN-β in response to lipotoxic stimulation [74]. Treatment with PPARγ agonists improved sepsis outcome, decreasing pro-inflammatory cytokines, increasing IL-10 levels, and improving mouse survival [73, 75]. In a recent study, a PPARγ response element was identified in the microRNA-124 (miR-124) promoter region, and expression of miR-124 was decreased in human sepsis patients and was critical for preventing an exaggerated inflammatory response in septic mice [72]. miR-124 expression was dependent on PPARγ signaling in both human THP-1 macrophage-like cells and RAW264.7 cells [72]. The enzyme PRMT1 was also recently shown to regulate PPARγ’s activity in macrophages in the cecal ligation and puncture sepsis models. Myeloid specific PRMT1 knock-out mice have higher pro-inflammatory cytokine production, defective PPARγ-dependent M2 macrophage activation, and lower survival rates [37]. Rosiglitazone treatment bypassed the IL-4/PRMT1 mechanism to restore PPARγ activity.

Other PPAR isoforms have also been linked to decreased severity of sepsis by down-regulating inflammation. In the cecal ligation and puncture rat sepsis model, treatment with a PPARβ/δ agonist inhibited signal transducer and activator of transcription 3 (STAT3) activation in AMs, reduced the inflammatory response, and prolonged rat survival [76]. However, bacterial counts in the lung were not lowered. Gene expression of the PPARα isoform was down-regulated in whole blood of patients with septic shock [77]. In the mouse model, PPARα signaling was key to amelioration of inflammatory cytokine production during sepsis. Cell surface markers of activation on splenic macrophages were reduced in PPARα-deficient, septic animals correlating with increased bacterial burden in the lung and splenic tissues [77]. Collectively, these data indicate that PPAR signaling is important for anti-inflammatory macrophage responses during sepsis and suggest that PPAR agonists could alleviate damaging inflammation associated with sepsis, improving disease outcome.

### Fungi and parasites

Fungi can also induce PPARγ expression and activity in macrophages. PPARγ ligands attenuate *Candida albicans* colonization of the gastrointestinal (GI) tract in mice in a dectin-1 dependent manner [78]. The antimicrobial peptide P17, isolated from ant venom, up-regulates PPARγ expression and activity in human macrophages, leading to increased expression of dectin-1 and MR and less severe *C*. *albicans* GI infection [79]. A subsequent study identified the NR liver receptor homologue-1 (LRH-1) as important for IL-13-induced PPARγ ligands that are critical for combating GI and systemic *C*. *albicans* infection [80]. Deficiency in LRH-1 in mice is detrimental to IL-13-mediated M2 macrophage polarization, which is needed to prevent host-damaging inflammation. This study identified a signaling pathway that uses at least two NRs for optimal macrophage responses against *C*. *albicans*.

PPARγ expression is also regulated by parasite infections [81–84]. During infection with *Plasmodium berghei*, malaria-susceptible mouse strains CBA and C57B6 had higher constitutive PPARγ levels compared to malaria-resistant BALB/c mice; however, in the susceptible mice, PPARγ did not translocate to the nucleus and the mice produced less plasmodicidal NO and H_2_O_2_ [82].

*Trypanosoma cruzi* infection increased PPARγ expression in murine peritoneal macrophages [83, 84]; however, *T*. *cruzi* induced PPARα but not PPARγ expression in BMDMs and RAW264.7 macrophages [85]. These data propose a site-specific role for PPAR signaling in macrophages in response to *T*. *cruzi* infection, similar to that observed for *M*. *tuberculosis* [61]. Both PPARγ and PPARα agonists induced M2 macrophage activation in peritoneal macrophages infected with *T*. *cruzi*, increasing phagocytosis of the parasites; however, the anti-parasitic activity of the macrophages was unclear [84, 85]. A new synthetic PPARγ ligand, HP24, decreased inducible nitric oxide synthase (iNOS) expression and stimulated pro-angiogenic mediators in macrophages and in the heart of *T*. *cruzi-*infected mice, reducing TNF-α and IL-6 release by macrophages [83]. Therefore, induction of PPARγ signaling appears to have a protective role during infection with *T*. *cruzi*. Administration of PPARγ agonists to *T*. *cruzi* patients may be a feasible approach to treatment.

PPARγ is also implicated in macrophage responses to schistosomes. Stimulation of peritoneal macrophages and BMDMs with schistosomal-derived lipids or purified lysophosphatidylcholine increased PPARγ expression and induced gene expression of the M2 macrophage hallmarks Arg1, MR, FIZZ1, TGF-β, IL-10, and prostaglandin E2 (PGE2) [86]. During *Schistosoma japonicum* infection in mice, pioglitazone treatment prevented hepatic and splenic pathologies, decreased Th1 T cells and increased regulatory T cells (Tregs) in the liver and spleen [87]. Furthermore, pioglitazone treatment of macrophages promoted Tregs whereas PPARγ inhibitor-treated macrophages reduced the proportion of these cells. The above studies provide evidence that PPARγ signaling is important for antifungal and antiparasitic activity of macrophages by regulating inflammation.

Overall, PPARγ down-regulates pro-inflammatory immune responses during infection, which is vital during the resolution phase to prevent inflammation-induced host damage (Fig 2). Use of PPARγ agonists or antagonists to treat infectious diseases must take into account the timing of administration and the pathogen and must balance pro- and anti-inflammatory mechanisms to enable an improved outcome. Targeting strategies for other PPARs await further studies.

### LXRs

LXRs regulate cholesterol homeostasis and transport and are regulated by oxysterols and intermediate products of cholesterol biosynthetic pathways [88, 89]. LXRs have two isoforms, LXRα (NR1H3) and LXRβ (NR1H2; Table 1), and form obligate heterodimers with RXR [90]. LXRs are also linked to M2 macrophages [91] and could be an attractive target for protection against disease (Fig 2).

### Bacteria

LXRs play a role in macrophage survival, preventing bacterial-induced apoptosis during infection with *Listeria monocytogenes*, *Bacillus anthracis*, *Escherichia coli*, and *S*. *Typhimurium* [92, 93] and contributing to bacterial clearance and resistance to *L*. *monocytogenes* infection. LXRs have also been linked to macrophage phagocytic responses during *S*. *Typhimurium* infection. LXR agonists reduced the intracellular levels of NAD in a CD38-dependent manner, counteracting *S*. *Typhimurium*-induced changes in macrophage morphology and distribution of F-actin in the cytoskeleton [94]. In the mouse model, LXR agonist treatment ameliorated clinical signs of *Salmonella* infection in a CD38-dependent manner [94].

LXRs are implicated in resistance to *M*. *tuberculosis* infection [64]. Macrophages infected with *M*. *tuberculosis* up-regulate LXRα [95] and LXRα deficiency leads to reduced control of intracellular bacterial growth [59, 96]. A build-up of lipid bodies in macrophages is associated with nonprotective responses to *M*. *tuberculosis*. LXRα activation in THP-1 macrophages increased the expression of the ATP-binding cassette transporters ABCA1 and ABCG1, which are responsible for lipid efflux, resulting in decreased lipid body formation during infection with attenuated *M*. *tuberculosis* strain H_37_R_a_ [95]. Administration of cholesterol-reducing statins has shown promise for tuberculosis (TB) treatment in animal models and clinical trials, which is curiously suggested to occur by reducing LXR activity [97–99]. Further evaluation of LXR activity concurrent with statin administration is required to discern how this intervention affects TB treatment as well as treatment of other infectious diseases, such as *S*. *aureus* [100], whose clearance can result from statin usage.

### Viruses

Up-regulation of the LXR effector ABCA1 is linked to suppression of HIV in humanized mice [101]. Pretreatment of human macrophages with LXR agonist TO901317 reduced the susceptibility of the macrophages to HIV infection, correlating with reduced lipid rafts and up-regulation of ABCA1 expression [102]. Prophylactic and therapeutic administration of the LXR agonist in mice increased resistance to HIV infection and reduced lipidemia [102]. Recent transcriptomic studies investigating susceptibility to dengue virus with a Cuban cohort of African ancestry revealed increased gene expression of RXR in individuals that were more resistant to infection and suggested a protective role for LXR/RXR against dengue virus [103].

LXR signaling was recently implicated in protection against gammaherpes virus MHV68, in which the virus up-regulated LXRα expression in a type-1 interferon dependent manner, which resulted in only moderate to no increase in gene expression for classic LXR target genes ABCA1, FADS2, and SCD2 [104]. Gene expression for ABCA1, FADS2, and SCD2 in macrophages was significantly increased in LXR knock-out mice prior to and after infection with MHV68 [104], leaving the authors to posit that LXR deficiency results in the modulation of LXR corepressor activity, thus increasing transcription of LXR target genes. Macrophages from LXR deficient mice had increased fatty acid and cholesterol synthesis, two metabolic pathways that support viral replication. Indeed, LXR deficient macrophages permitted increased replication of MHV68. Taken together, these data indicate that LXRs are important for antiviral activities, however the mechanisms of action appear to be distinct for each virus.

### Fungi and parasites

LXRs are beneficial against bacterial and viral infections [92, 94, 96, 104]. However, mice lacking LXR signaling are more resistant to infection with *Leishmania chagasi* and *Leishmania infantum* [105]. BMDMs from LXRα knock-out mice stimulated with IFN-γ had increased parasite killing ability compared to IFN-γ treated BMDMs from wild-type (WT) mice, producing more nitric oxide (NO) and IL-1β. In addition, LXR ligands abrogated NO production in WT macrophages in response to infection. These data indicate that LXR modulation of host antimicrobial responses is pathogen-specific. Further study is needed to unravel the complex signaling pathways involved.

### LXR regulation of macrophage inflammatory networks

Numerous mechanistic studies have been undertaken to understand the effects of macrophage LXR activity on inflammatory responses to microbes and their byproducts. Pathogens can interfere with cholesterol metabolism by inhibition of the LXR signaling pathway. For example, microbial ligands initiated interferon regulatory factor 3 (IRF3)-induced TLR3/4, blocking the induction of LXR target genes, including ABCA1, in cultured macrophages [106]. During zymosan-induced acute inflammation, enhanced signaling by the receptor tyrosine kinase Mer increased the activity of LXR, aiding in control of the inflammatory response in macrophages [107].

In multiple macrophage models, LXR activation resulted in down-regulation of LPS-induced inflammatory responses. Both LXRα and LXRβ are expressed in Kupffer cells [108] and required for Kupffer cell development [29]. However, LXRα/β knock-out mice have an increased number of hepatic mononuclear cells with macrophage surface markers that displayed an enhanced inflammatory response to LPS [108]. Pretreatment of Kupffer cells with an LXR agonist prior to LPS administration decreased the amount of pro-inflammatory cytokines produced and increased IL-10 production [109]. This study also suggested that LXRα competes with IRF3 for binding to glucocorticoid receptor-interacting protein 1 (GRIP1), repressing the transcriptional activity of IRF3 and NF-kB, thus inhibiting the LPS-induced inflammatory response. In murine BMDMs, LXRs down-regulate release of pro-inflammatory IL-18 due to LXR’s negative regulation of caspase 1 expression and activation [110]. LXR ligands also induced the expression of IL18BP, a potent IL-18 inhibitor, in an IRF8-dependent manner [110], demonstrating multiple mechanisms at play to limit IL-18 by LXRs.

Taken together, LXR activity generally appears to be an effective method to curb inflammation and induce protective immune responses against infections. However, like other NRs, LXR activity is not protective against all pathogen types. Additional work is needed to discern LXR’s capacity as an HDT for infectious and inflammatory conditions.

## Emerging NR-infectious disease interactions

### NR4A family

The NR4A family of nuclear hormone receptors is comprised of three members: Nur77 (NR4A1), Nurr1 (NR4A2), and Nor1 (NR4A3; Table 1). No endogenous ligand for these receptors has been identified, resigning the NR4A family to the orphan receptor category. However, interaction between Nur77’s ligand-binding domain and structurally diverse synthetic molecules have been observed and synthetic ligands have been identified for Nurr1 and Nor1 (reviewed in [111]). NR4As are involved in various cellular processes which are more extensively reviewed elsewhere [111, 112].

**Fig 3:**
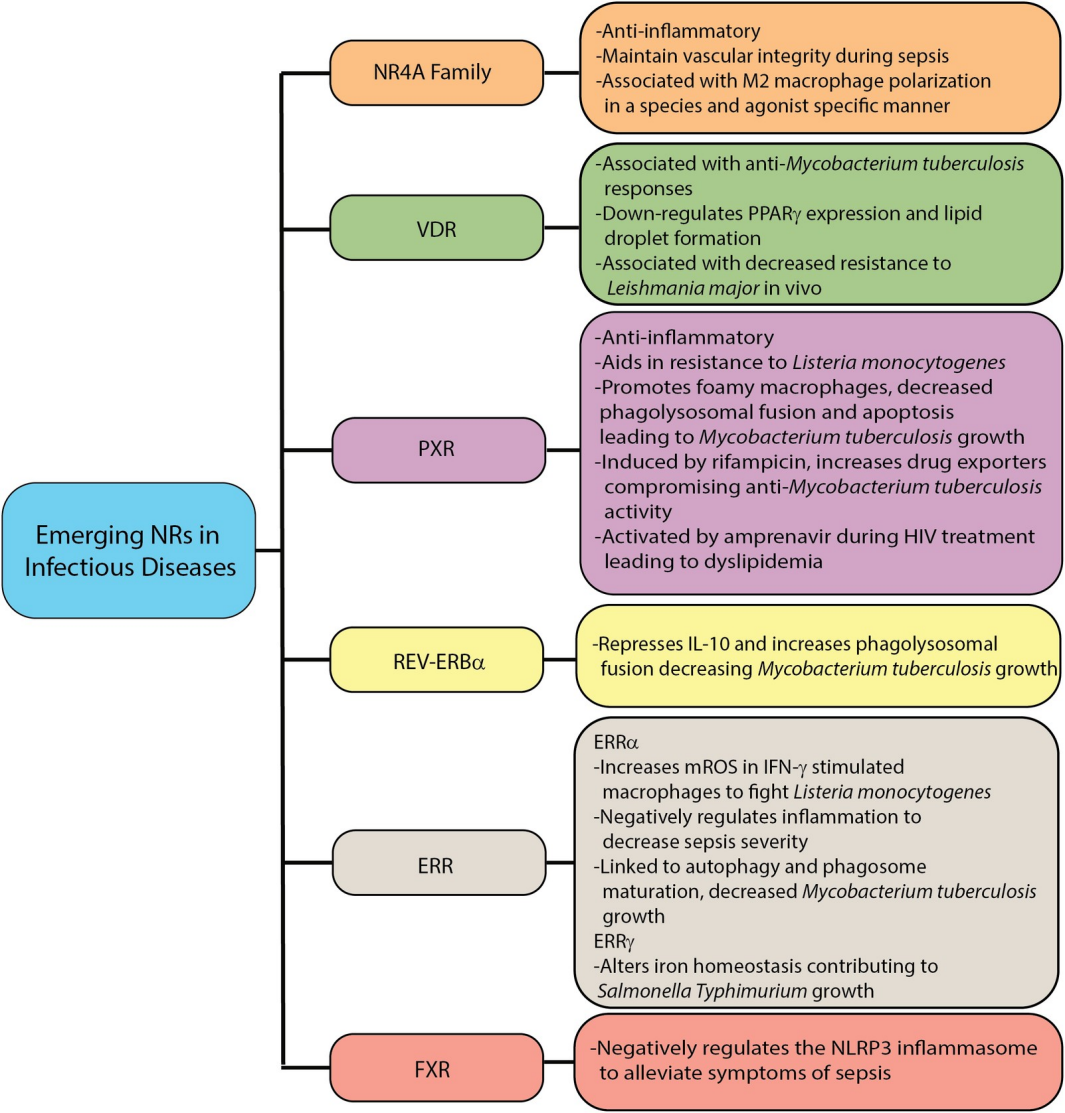
Newly described NR activity linked to macrophage responses to infectious diseases. Links between certain NRs and macrophage responses to infectious pathogens have recently emerged and are highlighted here. Overall, the NRs described here appear to aid in control of inflammatory responses of macrophages, leading to resistance against many diseases. The xenobiotic receptor PXR also induces efflux of antimicrobial drugs from macrophages, leading to dangerous side effects and susceptibility to *Mycobacterium tuberculosis* and HIV. Although no clear mechanistic links between these NRs and infection have been described, future study into these and other NRs may reveal novel targets for host-directed therapeutic development against infectious diseases. ERR, estrogen related receptor; FXR, farnesoid X receptor; IFN-γ, Interferon-γ; IL-10, interleukin 10; mROS, mitochondrial reactive oxygen species; NLRP3, NLR Family Pyrin Domain Containing 3; NR, nuclear receptor; NR4A, nuclear receptor 4A; PPARγ, peroxisome proliferator-activated receptor gamma; PXR, pregnane X receptor; VDR, vitamin D receptor.

All three members of the NR4A family are associated with anti-inflammatory responses because over-expression of these NRs decreased the production of pro-inflammatory cytokines and chemokines, whereas their knock-down resulted in increased cytokine/chemokine synthesis [45]. Nor1, but not the other NR4A NRs, is implicated in the generation of M2 human macrophages in response to IL-4 stimulation; however, gene expression for Nor1 was not increased in IL-4 stimulated murine BMDMs [47]. In contrast, overexpression of Nurr1 in murine BMDMs, but not Nor1 or Nurr77, was associated with M2 macrophage development by directly binding to the Arg1 promotor, conferring protection in an LPS-induced sepsis model [49]. LXR agonists induced Nor1 gene expression in murine Kupffer cells, which was important for LXR-mediated dampening of LPS-induced TNF-α production [113]. In an *E*. *coli*-induced peritoneal sepsis model, Nur77 did not play a role in host cytokine responses or leukocyte trafficking but was responsible for maintaining vascular impermeability, preventing distant organ damage [114] (Fig 3). Not much is known about NR4A family members for other infectious diseases. However, a recent study showed that murine pulmonary CD11c^+^ cells up-regulate Nur77 and Nor1 following *M*. *tuberculosis* infection with Nor1 being present in PBMCs from human TB patients [115].

Based on limited publications to date, it is apparent that gene expression and activity of NR4A family members vary with species and inflammatory stimuli. However, this family appears to be important for down-regulating inflammatory responses, which could be detrimental to fighting infectious diseases but may also be important for preventing host-damaging inflammation. Further investigation into the NR4A family’s role in macrophage responses to infection is necessary.

### VDR

The VDR (NR1I1; Table 1) is constitutively expressed in macrophages and has long been studied in conjunction with TB. Polymorphisms in the VDR gene have been associated with increased susceptibility to TB, however inconsistent results have been shown by meta-analysis (reviewed in [64]). Administration of vitamin D along with standard anti-TB drug regimens has improved clinical outcomes in some studies [116]. Addition of vitamin D to THP-1 macrophages down-regulated PPARγ and decreased the number of lipid droplets in *M*. *tuberculosis*-infected cells [117], demonstrating a role for the VDR in lipid metabolism during *M*. *tuberculosis* infection.

VDR has also been implicated in macrophage responses to parasitic infections. In a Columbian cohort, SNPs in the VDR gene may affect the immune response against *T*. *cruzi* [118]. In a mouse model of malaria, infection with *Plasmodium chaboudi* up-regulated VDR gene expression [119], suggesting that VDR may regulate malaria infection. VDR appears to be nonprotective during *Leishmania major* infection in mice, with disparity between mouse strains. VDR deficiency in the Th1-predisposed C57B6 mouse resulted in increased resistance to *L*. *major* whereas VDR knock-out BALB/c mice, which are Th2-biased, did not reveal a change in susceptibility (Fig 3) [120]. Further study is required to discern the role of VDR during parasitic infections.

### PXR

The human xenobiotic NR PXR (NR1I2; Table 1) is an adopted orphan NR that is predominantly expressed in the liver and intestines but is also expressed on monocytes and/or macrophages and lymphocytes [121]. PXRs inhibit both innate and adaptive immune responses [122, 123] and are also important for lipid uptake [124]. PXR deficient mice are highly susceptible to *L*. *monocytogenes* infection, in part due to excessive pro-inflammatory cytokine and chemokine production that resulted in death of inflammatory monocytes [123]. During *M*. *tuberculosis* infection, PXRs promoted foamy macrophage formation and decreased phagolysosomal fusion, inflammatory responses, and apoptosis in human macrophages, enhancing bacterial survival [125].

PXRs regulate various drug metabolizing enzymes and drug transporters that are vital for metabolism, detoxification, and elimination of xenobiotics [126]. Rifampicin, a first line anti-TB drug, is a potent activator of PXRs, which in turn compromises the effect of rifampicin in *M*. *tuberculosis*-infected human macrophages [127]. Protease inhibitors (PIs) used to treat HIV are associated with dyslipidemia and increased cardiovascular risk and several PIs activate PXRs. Amprenavir, a widely used PI in defense against HIV, is a potent PXR-selective agonist, inducing PXR-targeted CD36 gene expression in mice, resulting in dyslipidemia [128]. Additional studies are warranted to investigate the utility of PXR activity blockade in order to increase drug effectiveness while limiting side effects during treatment of infectious diseases.

### REV-ERBα

REV-ERBα (NR1D1; Table 1) is a unique NR with an atypical ligand-binding domain that lacks the carboxy-terminal activation function region responsible for transcriptional activation [129]. REV-ERBα is a constitutive transcriptional repressor and competes with other NRs (i.e., PPARγ and LXRs) for response elements, thus altering transcriptional activity [40, 130, 131]. Porphyrin heme was identified as the ligand for REV-ERBα in 2007 [132], changing its categorization to adopted orphan receptor.

The activities of REV-ERBα during infectious disease are mostly unknown, however recent studies with *M*. *tuberculosis* and LPS challenge have indicated a role for this NR during infection (Fig 3). In human macrophages, REV-ERBα knock-down led to decreased phagolysosomal maturation whereas treatment of THP-1 macrophages with a REV-ERBα agonist increased the number of autophagosomes and lysosomes, correlating with enhanced *M*. *tuberculosis* clearance [133]. Overexpression of REV-ERBα in human macrophages led to repression of IL-10 and increased phagolysosomal fusion and anti-*M*. *tuberculosis* activity [41]. This study revealed that a REV-ERBα binding site resides in the promoter region of IL-10 in both humans and nonhuman primates, but not in mice, demonstrating a species disparity [41]. REV-ERBα negatively regulated monocyte chemoattractant protein-1 (MCP-1) and TNF-α secretion in human macrophages stimulated with LPS [40]. REV-ERBα antagonism also suppressed IL-6 and CCL2 gene expression in mice during LPS endotoxin challenge [42, 43], revealing that this NR has both pro- and anti-inflammatory activities. Further investigation into REV-ERBα’s influence on macrophage responses to infection is required.

### Estrogen-related receptors

ERRs have three isoforms—ERRα (NR3B1), ERRβ (NR2B2), and ERRγ (NR3B3; Table 1)—all of which aid in regulating bioenergetic pathways (reviewed in [134]). ERRα’s up-regulation of mitochondrial ROS contributed to IFN-γ-induced antimicrobial responses to *Listeria* [135]. ERRα has been linked to the activation of autophagy and phagosomal maturation to aid in macrophage defense against *M*. *tuberculosis* [136]. On the other hand, ERRα acted as a negative regulator of TLR-induced inflammatory responses by inducing *Tnfaip3* transcription [137]. ERRα also negatively regulated macrophage inflammatory responses to endotoxin-induced septic shock through inhibition of NF-kB signaling [137]. Another isoform, ERRγ, may be important for *S*. *Typhimurium* growth through regulation of hepcidin and host iron homeostasis, because an ERRγ inverse agonist (a molecule that binds to the receptor resulting in activity converse to that of an agonist) reduced intramacrophage proliferation of *S*. *Typhimurium* [138]. Taken together, these data indicate that ERRs have both protective and nonprotective host properties for pathogens and more study is needed to discern if targeting ERRs would be an effective approach to antimicrobial and/or anti-inflammatory therapy.

### FXR

FXR (NR1H4; Table 1) is a metabolic NR that regulates bile acid, lipid, and glucose metabolism (reviewed in [139]) and has recently been linked to sepsis. FXR negatively regulates the NLRP3 inflammasome by physically interacting with NLRP3 and caspase 1, and FXR expression in mice is inversely related to endotoxic shock sensitivity [140]. However, agonism of FXR with GW4064 did not inhibit shock in mice. In another study, this same FXR agonist inhibited NLRP3 inflammasome activation in an FXR-independent manner [141]. These studies highlight that conclusions must be drawn cautiously when interpreting data using only agonists or antagonists, or knock-down or knock-out approaches.

## Perspectives and future directions

Considering the pivotal role that NRs play in macrophage responses, in particular their ability to control inflammatory and metabolic pathways, targeting these receptors in macrophages is a promising new HDT approach for infectious diseases. The NRs PPARγ and LXR have been most studied in regard to bacterial, viral, fungal, and parasitic infections; however, the importance of other NRs, including the NR4A family, PXR, and REV-ERBα is beginning to emerge (Fig 3). Given that 48 NRs have been identified to date in humans [6], it is likely that additional NRs will be identified to regulate macrophage responses to infection.

There are existing pharmacological interventions that target NRs, supporting their feasibility for use in combatting infectious diseases. However, it is clear that NRs modulate metabolism and inflammation in a tissue-, gene- and signal-specific manner. Furthermore, as highlighted in this review, certain NRs mediate either resistance or susceptibility to infection, depending on the microbe in question. Therefore, care must be taken when targeting specific NRs as a treatment for infectious diseases.

The next steps in the NR field require both a basic science and translational approach. It is critical that we identify the steps that occur upstream of NR activation. Cross-talk between NRs as well as between NRs and the signaling pathways of PRRs including TLRs, nucleotide oligomerization domain (NOD)-like receptors (NLRs), and C-type lectin receptors (CLRs) must be defined to discern the mechanisms behind NR activation. Further understanding regarding ligands (endogenous and synthetic) for NRs and NR effectors is also required. In addition, it is imperative to consider the translatability between in vitro and in vivo systems as well as potential species disparities. Further research into these topics will be key for identification of NRs that can be used to treat infectious diseases in humans.

Some NR agonists and antagonists lack specificity for their intended target, resulting in off-target effects that can have undesirable or unacceptable side effects [142–144]. Therefore, future NR-targeted interventions will need to be more specific and studied carefully in different infectious disease models. For example, many NRs are involved in the inflammatory response during infection, making NRs attractive targets to limit tissue-damaging inflammation. However, the timing of administration will need to be investigated further so as to not hinder the natural host antimicrobial response. Furthermore, the broad and diverse roles of NRs including regulation of basic processes like circadian rhythm and cholesterol transport or their roles in other diseases such as autoimmunity, obesity, and atherosclerosis must be taken into account when using NR modulators to treat infections.

We must also be cautious when drawing conclusions based on agonist–antagonist data. Both agonists and antagonists should be used and knock-down and/or knock-out models, when available, should also be used to confirm that the effects observed with agonists–antagonists are specific and not due to off-target effects. Similarly, concentration and timing of agonist–antagonist administration should be carefully planned to reduce likelihood of undetected off-target effects.

In conclusion, there is a need to continue to study NR mechanisms of action in well-defined model systems in order to develop new chemical classes of ligands and inhibitors with the ability to selectively modulate NR function. This selectivity of novel HDTs must be achieved if this line of treatment is to be feasible. In this era of increasing antimicrobial resistance, future study identifying and characterizing NRs during infections is not only warranted but compulsory if we are to consider new HDT approaches that augment antimicrobials in combating these deadly diseases.
